# Modelling the wind potential energy for metallurgical sector in Albania

**DOI:** 10.1038/s41598-024-51841-x

**Published:** 2024-01-14

**Authors:** Klodian Dhoska, Elena Bebi, Irida Markja, Parid Milo, Ermil Sita, Serxhi Qosja

**Affiliations:** 1 Department of Production and Management, Faculty of Mechanical Engineering, Polytechnic University of Tirana, Square Mother Theresa No. 1, 1019 Tirana, Albania; 2 Albanian Mechanical Engineering Association (AMEA), Mother Theresa Square No. 1, 38954 Tirana, Albania; 3 Association of Talent Under Liberty in Technology (TULTECH), Sopruse pst 3, 10615 Tallinn, Estonia; 4 Department of Energy, Faculty of Mechanical Engineering, Polytechnic University of Tirana, Square “Mother Theresa” No. 1, 1019 Tirana, Albania; 5 Department of Mechanics, Faculty of Mechanical Engineering, Polytechnic University of Tirana, Square “Mother Theresa” No. 1, 1019 Tirana, Albania; 6 Everest Ltd, Metallurgical Industry, Bulevardi Blu 544, Kamëz, 1030 Tirana, Albania

**Keywords:** Energy science and technology, Engineering

## Abstract

The metallurgical industry, in the context of the global energy crisis and the new European green deal, needs urgent investments on energy and resource efficiency. The metallurgical sector, which includes the production of different metals is an energy-intensive industry that requires large amounts of energy for various processes such as smelting, refining, and casting. One of the largest consumptions of energy in Albania comes from the metallurgical sector during the production of iron, steel, chromium and aluminum which corresponds respectively to three private companies called “Kurum International Ltd”, “AlbChorme Ltd” and “Everest Ltd”. During the last three years, these companies have temporary interrupted the production process due to the higher electricity price that come from imports. Based on it, our research work presents the energy efficiency analysis in the Albanian metallurgical sector by focusing on the implementation of wind energy in the above mentioned private metallurgical companies, because adding new generation capacity from Renewable Energy Sources in a context of industrial energy communities, will contribute to improve the security of supply for this industry. The Wind Balkan Atlas, New European Wind Atlas, and Wind Atlas Analysis and Application Program (WAsP) has been used to select the appropriate areas and to develop the wind potential distribution maps, as well as to select the most suitable type of wind turbine based on capacity factors. Two areas were selected close to the metallurgical sectors in the regions of “Vajkal” in Bulqizë and “Selitë e Malit” in Tirana. It has been installed the power of 9 MW for each wind farm, with a capacity factor of 40% and 36.6% respectively, and with a total annual energy production of about 60 GWh/year, these wind farms will cover about 26% of the total annual consumption of companies. Clean Energy Management Software (RETScreen Expert) was used for the detailed economic analysis and environmental impact of proposed wind farms. The economic sensitivity analysis of the proposed wind farms showed that even for the highest installation cost value of 1350 €/kW, for discount rates 5, 7, and 11%, the *LCoE* values are within the statistically established range for wind farms in Europe.

## Introduction

In recent years, there has been a growing trend towards the use of renewable energy sources and energy efficiency measures in the metallurgical sector to reduce greenhouse gas emissions and mitigate climate change. The metallurgical sector, which includes the production of iron, steel, aluminum, copper, and other metals, is an energy-intensive industry that requires large amounts of energy for various processes such as smelting, refining, and casting. The energy systems in the metallurgical sector can be categorized into two types: primary energy systems and secondary energy systems. Secondary energy systems involve the use of by-products from primary energy systems or renewable energy sources such as hydropower, solar and wind energy. Furthermore, energy efficiency in metallurgy sector includes an implementation of energy-efficient technologies such as optimization of incoming energy and materials flows, waste heat recovery, energy-efficient lighting and equipment^1–3^.

The metallurgical sector in European Union uses up to 33% of the total energy consumptions that comes from productions of ferrous and non-ferrous metals^2,4,5^. Based on it several strategies in many developed countries have been implemented in the metallurgical sector to reduce the usage of primary energy and materials, such as optimization of incoming energy and materials flows, adjustment of energy-related processes, and valorization of process residues^6–8^. Furthermore, it has been adopted renewable energies for supporting metallurgical sector and reducing environmental impacts^9–11^.

According to the latest available data from the International Energy Agency (IEA), the industrial sector in Albania accounted for approximately 28% of the country's total energy consumption^12^. This includes energy used in metallurgy, manufacturing, construction, mining and other industrial activities. The largest energy-consuming sub-sectors are coming from the production of iron, steel, ferrochrome and aluminum from Albanian metallurgical companies called respectively “Kurum International Ltd”, “AlbChorme Ltd” and “Everest Ltd”. Albania’s energy system is primarily based on hydroelectric power and some smaller amounts of thermal energy. Hydroelectric power accounts for approximately 95% of Albania's electricity generation, with the remaining 5% coming from solar energy. In general, the energy system covers 60% of energy consumptions and the other 40% comes from imports. In the current conditions of the global energy crisis, the import of electricity in Albania means higher costs of products and services for all consumers in the country. During the Covid-19 pandemic, the energy consumption in Albania from the metallurgical sector surpassed the residential consumption^13^. As a result of the high prices of imported electricity, the two aforementioned companies have stopped their activity. Due to the high level of energy consumption that comes from metallurgical sector as well as suitable climate conditions the Albanian government has given support to private companies for increasing and implementing renewable energies by focusing on hydroelectrically power, solar and wind energy. Furthermore, a sustainable development of the energy sector in particular for the metallurgical sector, in the conditions of our country, requires the diversification of renewable energy sources by implementing wind and solar energy on a large scale. This way, the energy needed to produce materials and make them into products, would be mostly coming from renewable energy, thus reducing the carbon footprint in the life cycle of the products. Furthermore, renewable energies would reduce the environmental impact from thermal electricity import and decrease the number of the small hydropower plants.

In this paper, we present the energy efficiency analysis in Albanian metallurgical sector by focusing on the implementation of the wind energy in the above mentioned private metallurgical industries. Modern-Era Retrospective Analysis for Research and Applications (MERRA), ERA5, New European Wind Atlas (NEWA) and Wind Balkan Atlas (WBA) has been used in most of the studies as the best tools to simulate wind power production^14–17^. WBA and NEWA has been used in our research work to select the appropriate areas. The Wind Atlas Analysis and Application Program (WAsP) was used to develop the wind potential distribution maps, as well as for selecting the most suitable type of wind turbine based on capacity factors. Clean Energy Management Software (RETScreen Expert) was used for the detailed economic analysis and environmental impact of proposed wind farms.

## Methodology and measurement results

**Figure 1:**
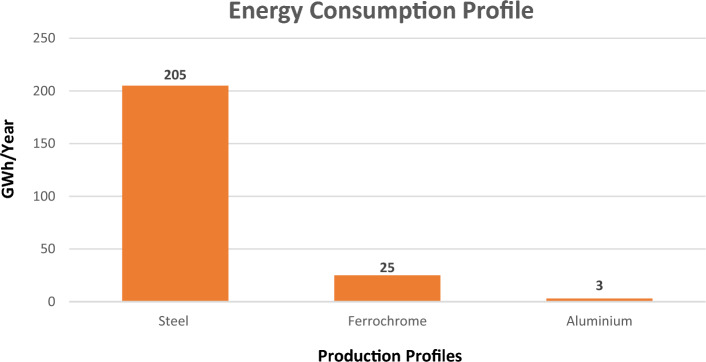
Energy consumption profile for the steel, ferrochrome and aluminum companies, located in Albania.

Under the context of the global energy crisis, the price increase in electricity and imports caused some Albanian metallurgical companies to temporarily interrupt parts of their activities. The energy consumption profile for these three metallurgical companies are shown in the Fig. 1.

This event emphasizes the need for higher energy security in Albania overall, and especially for the metallurgical sector. This would require diversifying the source of energy production through renewable sources, which for Albania means implementing solar and wind power. Some important factors mentioned below has made our research work to focused on the implementation of wind energy instead of solar panels:

- Producing energy from photovoltaic panels require a large surface area which is not ideal considering Albania is a small country with area of 28,748 km^2^.
- The production capacity of photovoltaic panels is small and insufficient to fulfill the needs of the metallurgical industry sector.
- There are higher costs associated with producing energy from photovoltaic panels compared to wind turbines
- The suitable areas for photovoltaic panels are predominantly used for agriculture and based on Albanian law they cannot be substituted for energy production.

**Figure 2:**
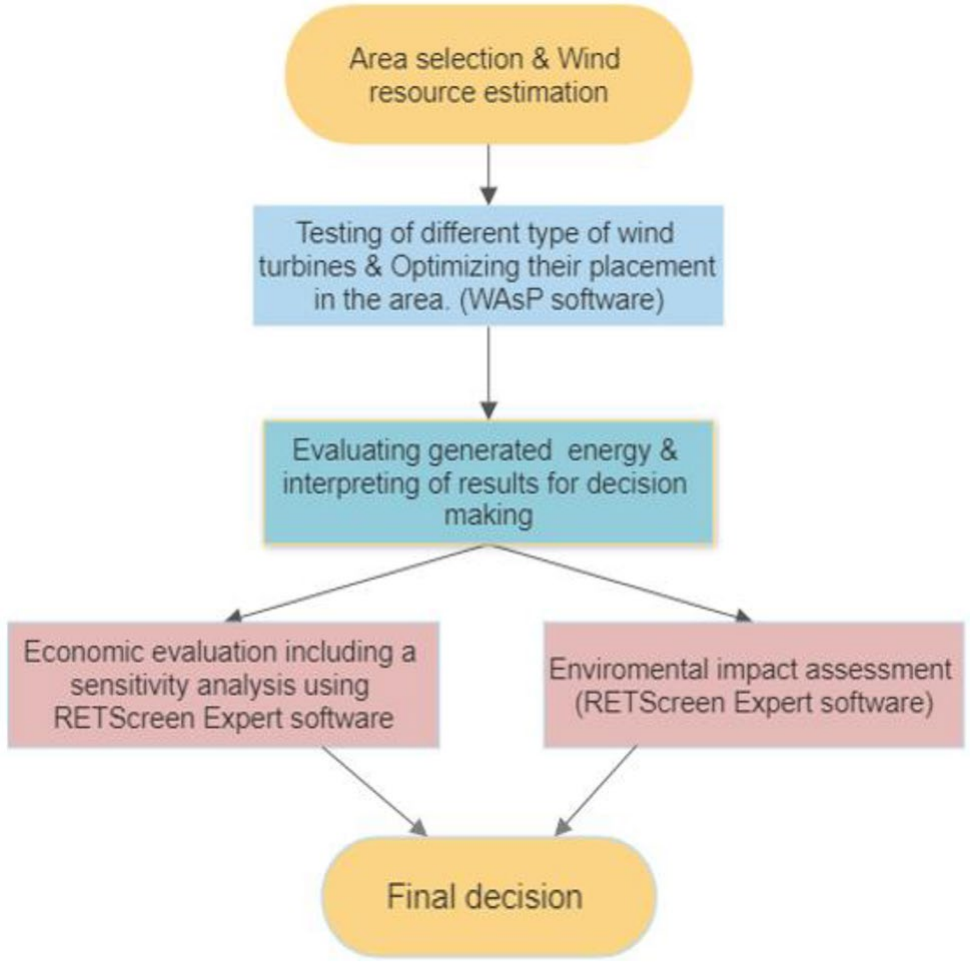
The flowchart of the feasibility study for setting up wind farms in the selected areas.

Two wind farms were simulated and proposed based on wind turbine placement that optimizes electric energy production and minimizes wake losses. Figure 2 depicts a methodology algorithm used for feasibility study of setting up wind farms in the selected areas.

### Areas selections

One of the reasons Albania has been slow to adopt wind power solutions, is because there is a lack of long-term measurements of wind speed in the country that follow the standards for selecting high potential areas for wind farms. Due to the lack of real measurements a good solution for identifying general areas of high wind potential are wind atlases. Many research works have shown that wind atlases are useful for initial assessment of wind conditions^18–23^.

**Figure 3:**
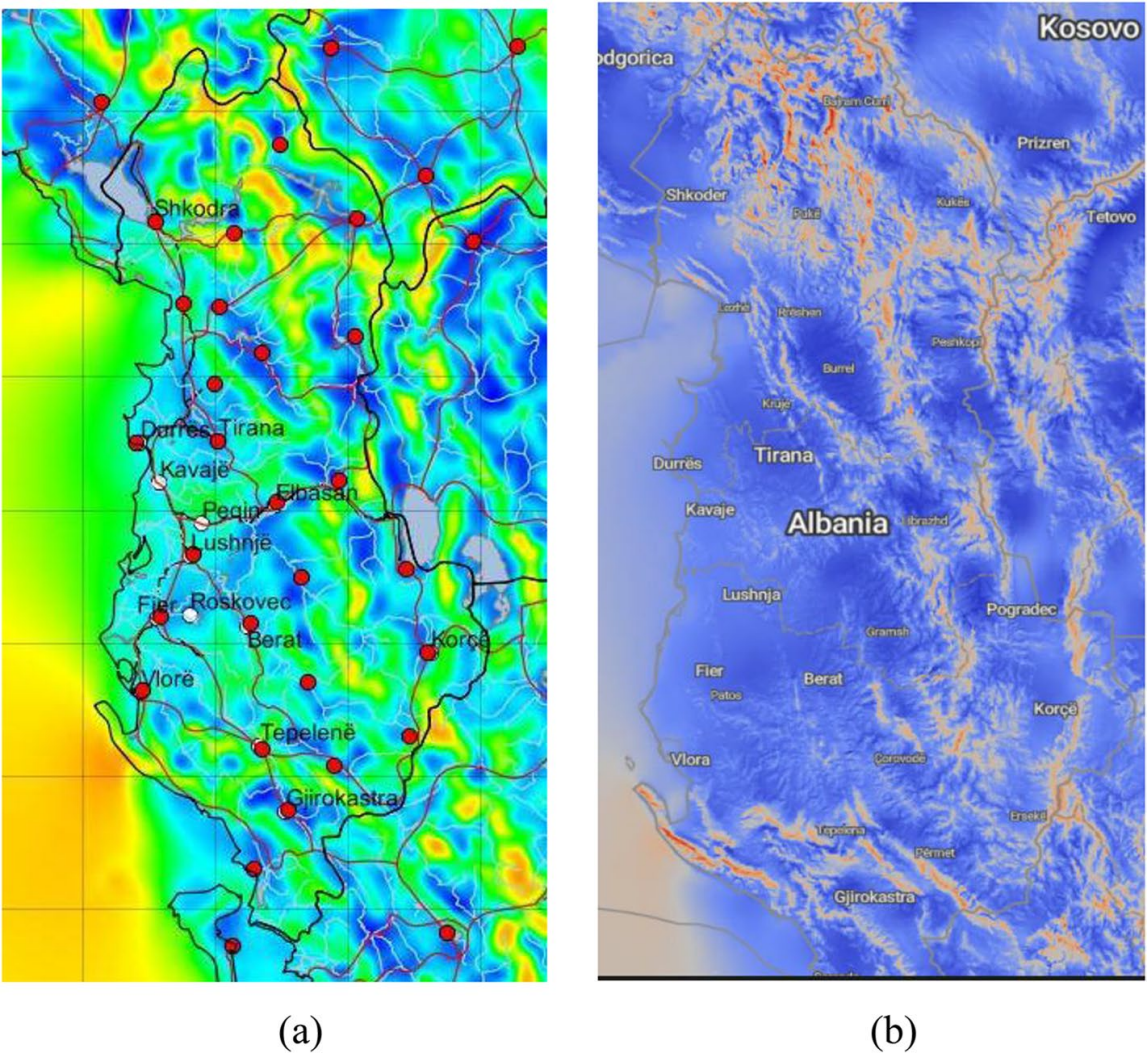
The selected areas in the map of wind speed distributions at 50 m a.g.l. in the Albania’s territory from both atlases: (**a**) Wind Balkan Atlas and (**b**) New European Wind Atlas.

Since 2019, Albania is specifically included in the Balkan Wind Atlas (BWA) as well as the New European Wind Atlas (NEWA). The business activities of the companies “Kurum International”, “Everest Construction Group”, and Ferrochrome Metallurgical Plant-Burrel, are located in central Albania. Wind speed distribution maps at 50 m a.g.l. from both atlases were used to investigate areas of high wind potential in central Albania, see Fig. 3. Important factors in selection were access to road infrastructure and transmission lines, land ownership and respecting forest areas. Therefore, geographic information system (GIS) was used since it offers information on topography, road infrastructure, transmission lines, land usage, formal and informal urban regions. GIS information is updated based on satellite images.

**Figure 4:**
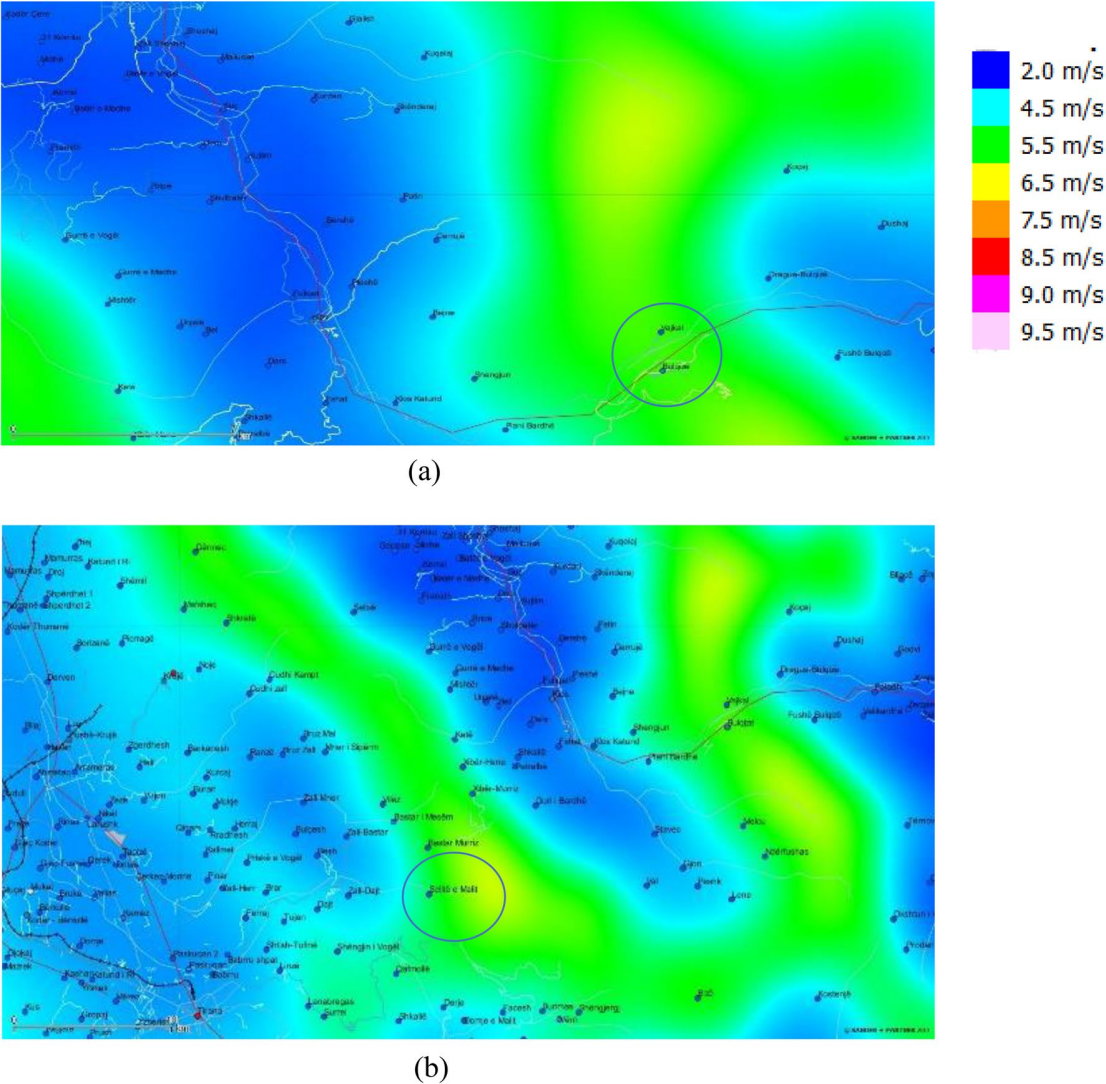
The selected areas in the map of wind speed distributions at 50 m a.g.l. from Wind Balkan Atlas: (**a**) “Vajkal”, Bulqizë and (**b**) Selitë e Malit, Tirana county.

The process of area selection is one of the most important aspects of planning and developing a new wind farm. After careful investigation based on wind potential, two areas were selected in the regions of “Vajkal”, Bulqizë and “Selitë e Malit”, Tirana county based on long-term data from BWA and NEWA, see Fig. 4. Long-term data on wind speed is crucial in forecasting long term energy production^14^. The Universal Transverse Mercator (UTM) coordinates of the hypothetical anemometric towers from both atlases for the selected areas are given in Table 1.

**Table 1:** UTM coordinates of hypothetical anemometric towers from the BWA and NEWA.

Areas name	BWA tower	NEWA tower
Vajkal, Bulqizë	North 4,596,386 m East 435,449 m	North 4,597,618.69 m East 434,585.32 m
Selita e Malit, Tirana	North 4,586,135 m East 417,274 m	North 4,586,420 m East 418,179 m

**Figure 5:**
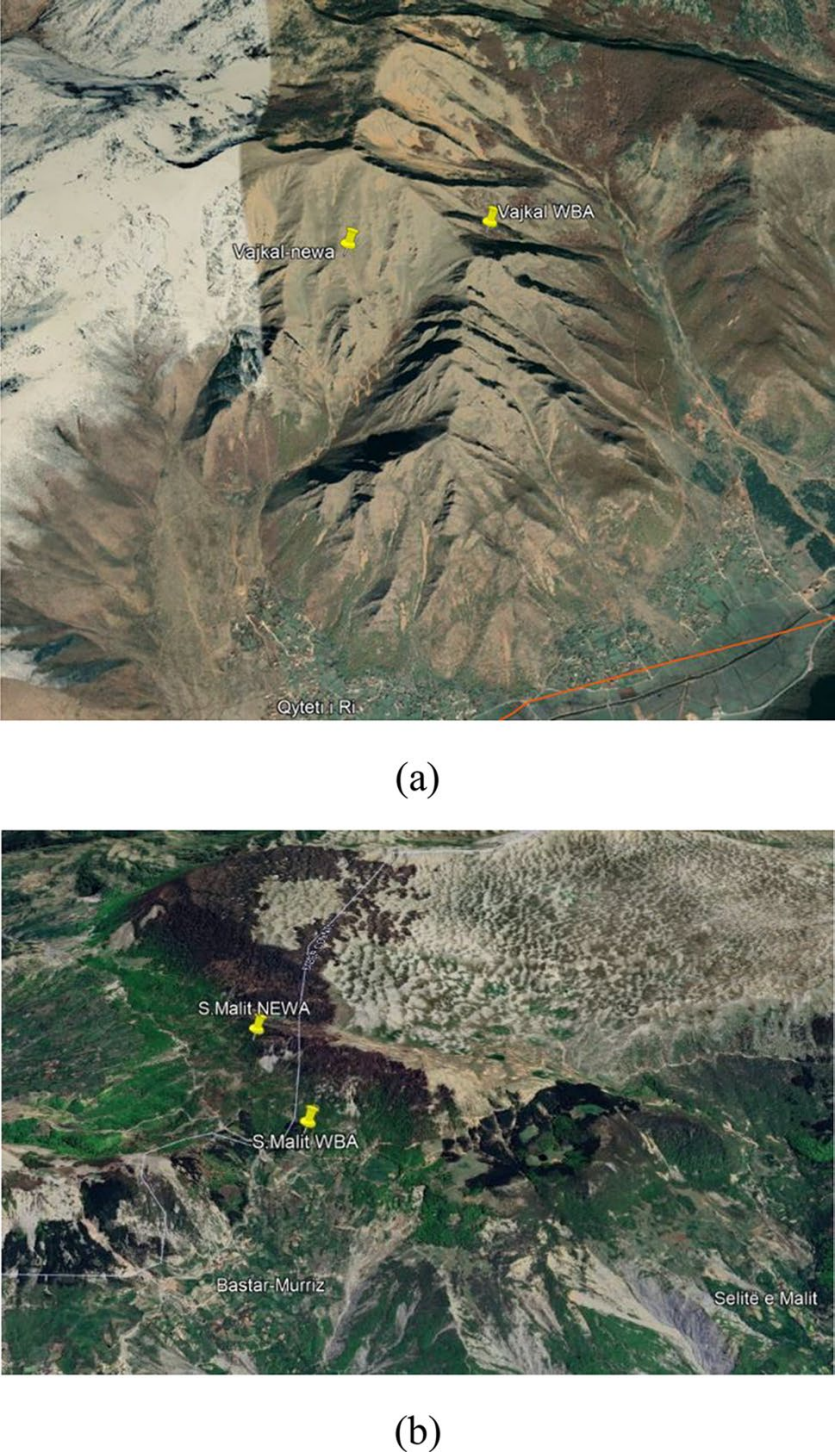
The mutual positions of the anemometric towers according to the two atlases: (**a**) in the area of “Vajkal”, Bulqiza and (**b**) “Selitë e Malit”, Tirana county.

Figure 5 depicts the reciprocal position of anemometric towers found in both atlases of the selected areas.

**Figure 6:**
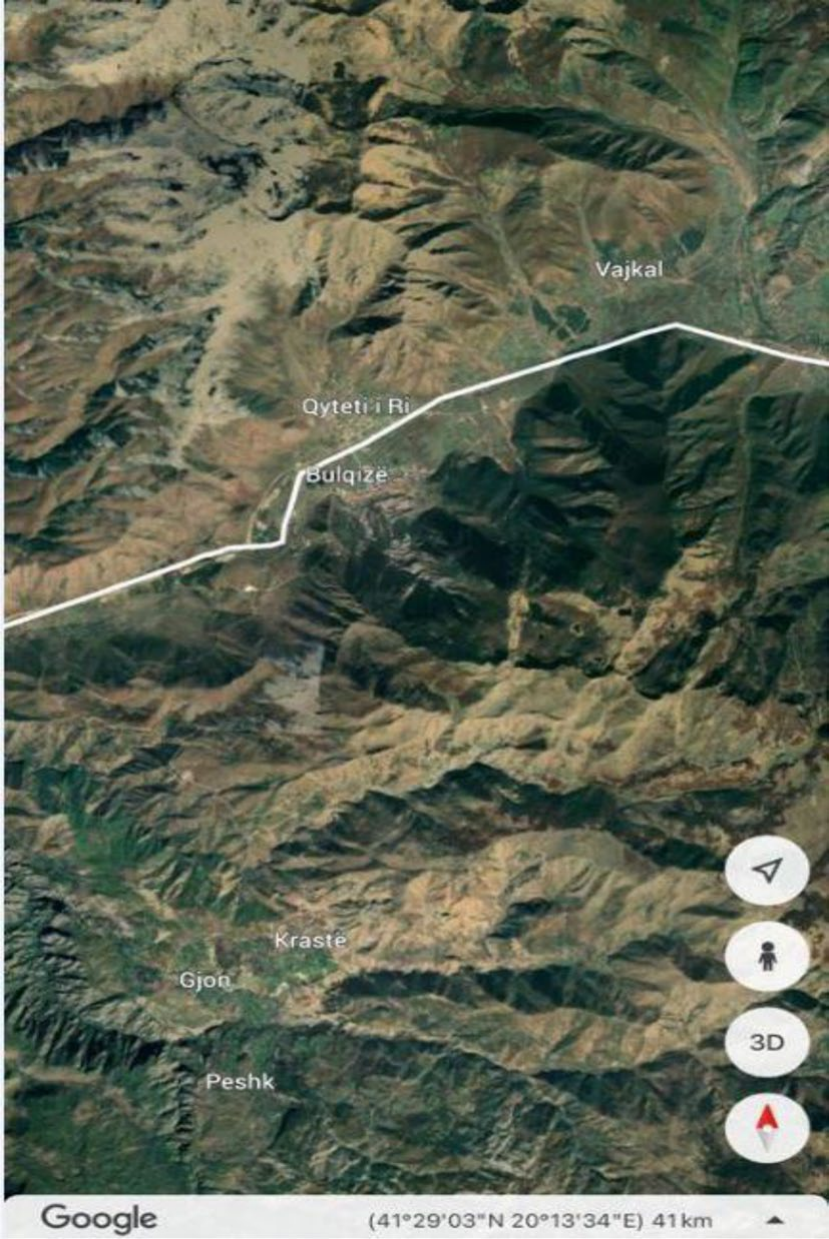
The white line shows the 110 kV voltage line and the Peshkopi-Maqellare national road that passes near the “Vajkali” area.

The terrain in both selected areas is land owned by the state. The “Vajkal” region includes a mountain slope over the town of “Vajkal” close to a transmission line (110 kV) and the national road Peshkopi–Maqellare, see Fig. 6. In case of “Selite e Malit” region, the top of mountain bare was thought to be the most suitable area without any environmental impact.

### Analysis of wind source for selected areas

**Figure 7:**
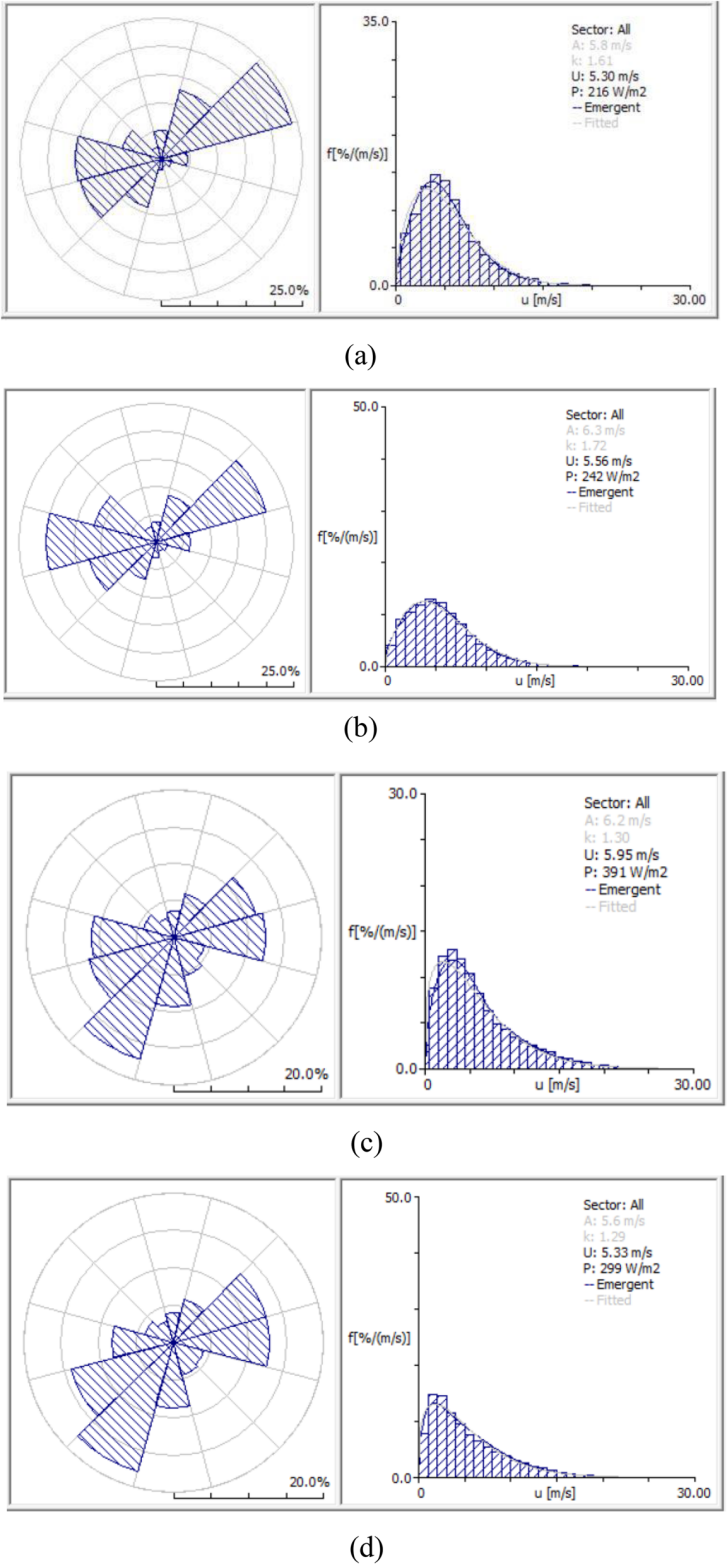
Wind rose charts and Weibull probability distributions for two selected areas at 50 m above ground level through WAsP. “Vajkal”, Bulqizë according to (**a**) BWA and (**b**) NEWA. “Selitë e Malit”, Tirana county according to (**c**) BWA and (**d**) NEWA.

Since the variation in wind speed impacts the wind turbines’ electric power production level, in order to achieve a credible mean yearly wind speed value, long term data over the span of 14 years at 50 m of altitude a.g.l. from both atlases (Balkan Wind Atlas 2000–2013 and New European Wind Atlas 2005–2018) were used for the selected areas. In order to determine the source of wind in the selected areas, the evaluation of the wind speed direction and power was performed using WAsP software. WAsP Observed Wind Climate (OWC) files for each atlas tower at 50 m above ground level were used to visualize the wind patterns by giving the wind rose charts and Weibull probability distributions as can be seen in Fig. 7.

The probability of a specific wind speed value is described in terms of the wind power density probability function. The Weibull probability distribution gives a very close estimate of the observed wind speed probability and is mathematically expressed with Eq. (1):

1 $$f\left(U\right)=\left(\frac{k}{A}\right){\left(\frac{U}{A}\right)}^{k-1}exp\left(-{\left(\frac{U}{A}\right)}^{k}\right) \quad \mathrm{ for }\,\,U>0\,\,\mathrm{ and }\,\,k, \,\,A > 0$$

where *A* (m/s) is a scale parameter, closely related with mean wind speed *U*, and *k* as a shape parameter is a measure of the width of the distribution. Higher values of *k* indicate a narrower wind speed range.

The comparison of wind rose charts of each area shows that the dominating wind blowing directions are very similar across both atlases. There are however some divergent values in mean wind speed, wind power density and in the *A* and *k* parameters of the Weibull probability distribution. Nonetheless, the values of parameters *A* and *k* as well as the mean speed *U* and power density *P* which result from both hypothetical anemometric antennas in each area show that these areas have a wind source which can be deemed adequate for setting up wind farms. Furthermore, in two previous research works^17,19^ it was shown that the models in both atlases are comparable to each other, but the New European Wind Atlas is closer to the real area measurements. Therefore, wind speed and power density distribution maps were developed with the WAsP program using NEWA’s OWC files.

**Figure 8:**
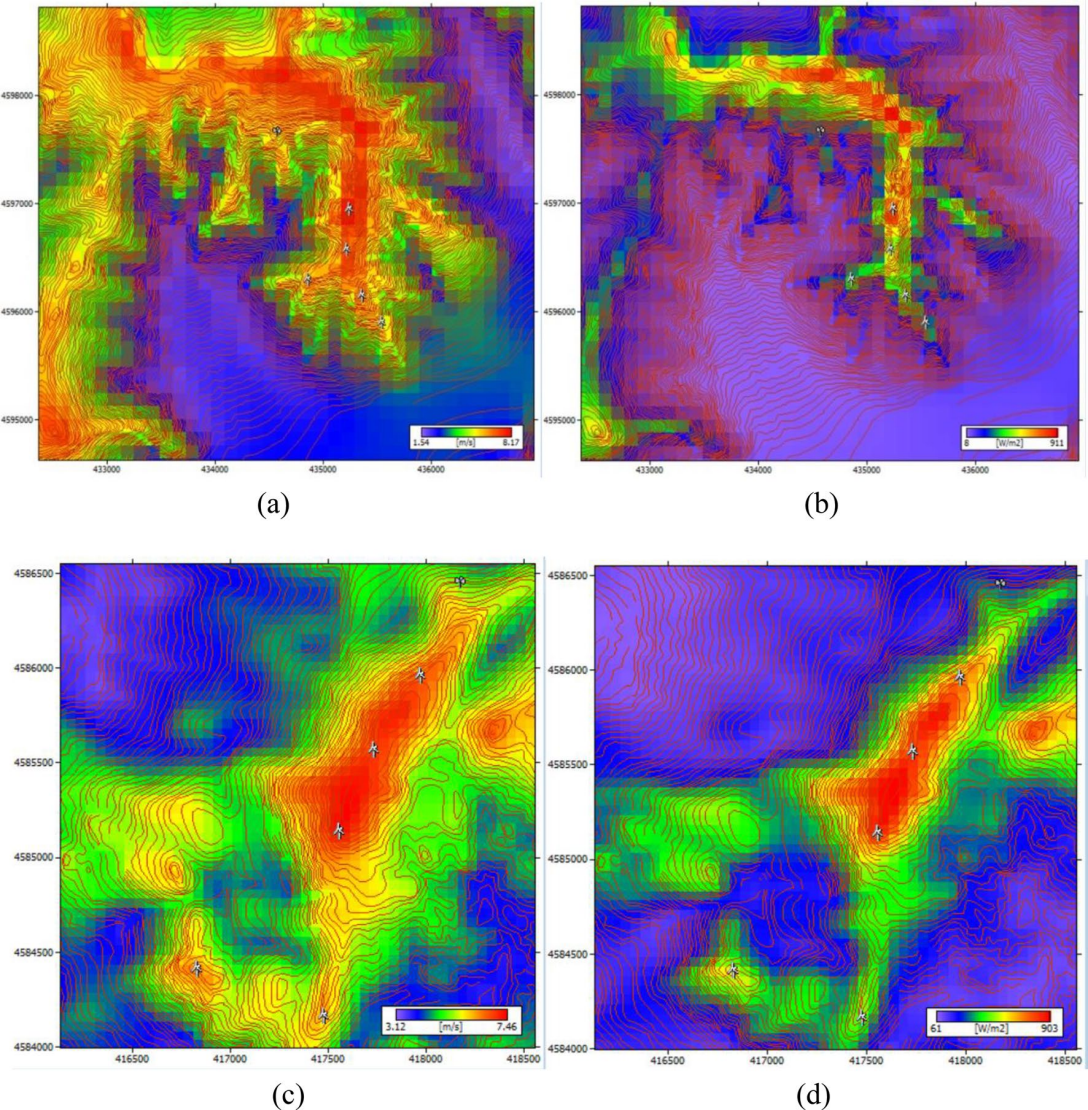
Wind potential distribution maps in selected areas at 50 m above ground level through WAsP. “Vajkal”, Bulqizë according to (**a**) wind speed and (**b**) power density. “Selitë e Malit”, Tirana county according to (**c**) wind speed and (**d**) power density. *Source*: Author’s design.

In the Fig. 8 are shown the wind potential distribution maps for both the selected areas, in terms of wind speed (m/s) and power density (W/m^2^) according to data from NEWA towers.

Table 2 depicts the mean speed (m/s) and power density (W/m^2^) in the areas with the highest wind speed potential in the two territories under study based on the wind potential distribution maps in the area developed by the WAsP simulation program.

**Table 2:** Mean wind speed (m/s) and power density (W/m^2^) at 50 m a.g.l. in selected areas according to data of period time 2005-2018 from NEWA.

Areas name	Mean wind speed (m/s)	Power density (W/m^2^)
Vajkal, Bulqizë	8.17	911
Selita e Malit, Tirana	7.46	903

**Figure 9:**
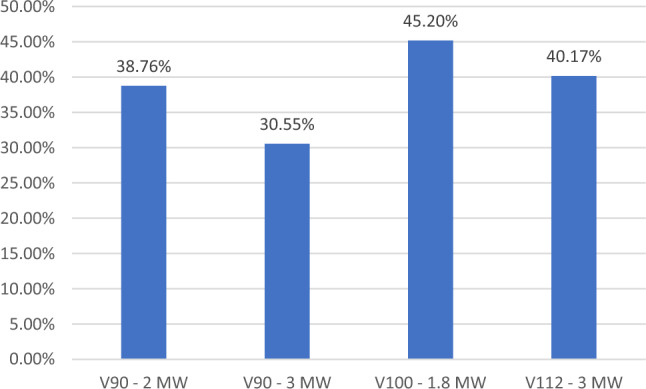
Capacity factors in the percentage for four different types of Vestas wind turbines tested in Vajkal, Bulqizë site.

Based on the wind speed class ranking, referring to speed values (m/s) and power density (W/m^2^) at 50 m of altitude a.g.l. both selected areas can be classified as wind power class IV, V and VI. Four different types of Vestas wind turbines were tested in the same wind source conditions. Currently there is a tendency towards larger turbines, with 2–5 MW rated energy generation, with rotor diameters that surpass 100 m and hub heights of 100–120 m^20^. In the Fig. 9 it has been shown that the turbine with the highest capacity factor was Vestas V100-1.8 MW^21^. The main characteristics of the selected wind turbines are presented in Table 3.

**Table 3:** The main characteristics of the selected wind turbines. *Source*: WAsP wind turbine files.

Model	Hub height (m)	Rotor diameter (m)	Cut-in wind speed (m/s)	Rated wind speed (m/s)	Cut-off wind speed (m/s)
V90-2.0	80	90	3	13.5	25
V90-3.0	80	90	4	15	25
V100-1.8	80	100	3	12	20
V112-3	85	112	3.5	15.5	25

Consequently, the WAsP program was used to develop the wind farm set up with a site layout design of 5 wind turbines V100-1.8 that maximizes annual electric power production and minimizes wake losses. The WAsP simulation program is a software that calculates a regional wind atlas and a resource grid in order to establish wind farms by performing a wind source analysis based on long term anemometric tower data. Table 4 shows the electric power production, wake loss, mean wind speed, and power density data applicable in the two parks proposed for “Vajkal” and “Selite e Malit”. The values were calculated using the WAsP simulation program.

**Table 4:** Data for the generated energy, wake losses, mean wind speed, power density, capacity factor for the proposed wind farms.

	Total	Mean	Min	Max
Vajkal, Bulqize				
Total gross AEP (GWh)	32.173	6.435	5.089	8.130
Total net AEP (GWh)	31.571	6.314	5.072	8.059
Proportional wake losses (%)	1.87		0.33	3.86
Mean wind speed (m/s)		6.72	5.71	8.11
Power density (W/m^2^)		432	292	700
Capacity factor (%)	40			
“Selite e Malit”, Tirana county				
Total gross AEP (GWh)	26.283	5.257	4.822	5.649
Total net AEP (GWh)	25.712	5.142	4.667	5.58
Proportional wake losses (%)	2.17		0.49	4.74
Mean wind speed (m/s)		6.09	5.56	6.48
Power density (W/m^2^)		459	319	540
Capacity factor (%)	32.6			

**Figure 10:**
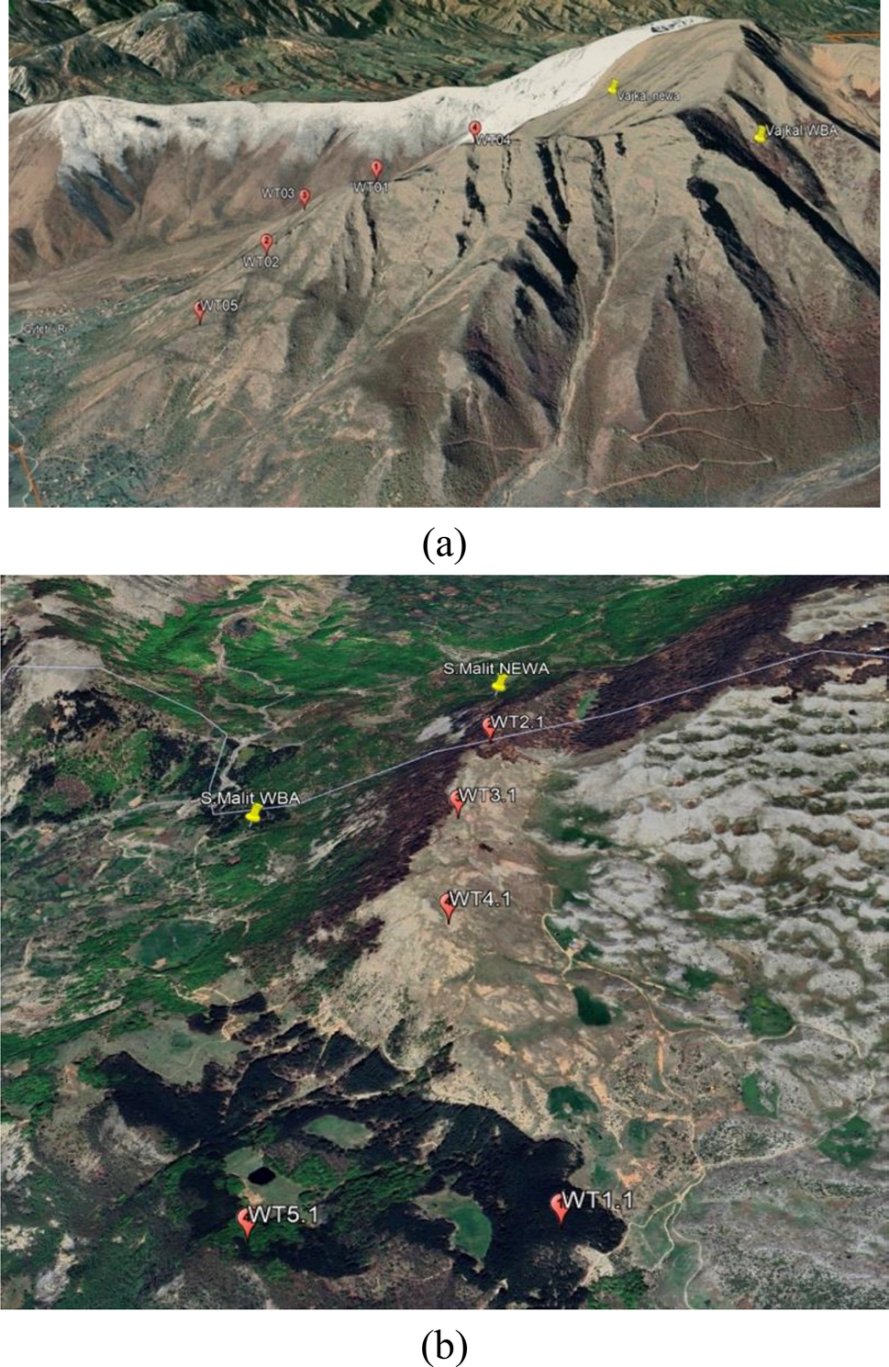
General view of proposed wind farms. (**a**) “Vajkal”, proposed wind farm and (**b**) “Selitë e Malit” proposed wind farm.

The area with high wind potential in the proposed "Selitë e Malit" park is in the top of the mountain which is bare of vegetation. While the plateau at the foot of the mountain is wooded. Therefore, the turbines were placed in the top of the mountain. In the Fig. 10 it can be shown an important overview in regarding the visual and environmental impact of placing the turbines in the proposed areas.

### Economic analysis

The decision to implement the proposed research work wind farm in the “Vajkal” and “Selite e Malit” areas will depend on the results of a detailed economic analysis.

### Wind energy costs

The costs that generate the implementation of wind farms are determined by several important factors related to the source of the wind, the efficiency of the wind turbines, lifetime of wind farm, the time that wind turbines are able to generate electricity, site layout design, capital costs, financing costs, as well operation and maintenance costs or variable costs. The capital costs of wind energy projects or upfront capital cost are often referred to as capital expenditures (CAPEX) and are the costs that dominate in wind energy projects^22^. They consist of the cost of the turbines, their transport and installation, cost of grid connection, the construction cost including foundations, roads and buildings, underground cabling within the wind farm, as well as the licensing procedural costs, long term measurement costs and preliminary design of the wind energy project. While these costs vary based on the types of turbines, markets and locations where they are placed, they represent 80% of the total cost of the project over its entire lifetime. The largest cost component is the cost of the wind turbines.

Wind turbine prices have declined globally over the last decade in spite of the continued increase in rotor diameters, hub heights and their rated powers. Since 2019 Vestas wind turbines continue to be under 1000 €, varying between 780 and 960 €/kW^22,23^. This has led to a decline in wind energy project capital costs regardless of some variability within the European Union. According to a report by the European Wind Energy Association (EWEA), the cost of installed power of onshore wind projects in Europe, differs between countries and typically varies from around 1100 €/kW to 1350 €/kW^24,25^. In 2019, the CAPEX was on average 1300 €/kW. The global weighted average total installed cost of onshore wind according to IRENA (2023) fell by 35% between 2010 and 2021, from 2042 to 1325 €/kW^22^.

For the economic sensitivity analysis of the two projects in our study, the total installed capital cost was assumed to have the values of 1100 €/kW, 1200 €/kW, 1300 €/kW, and 1350 €/kW. The Variable costs of an onshore wind project consist of operating & maintenance (O&M) cost, land rental, insurance, taxes, and administration cost.

O&M costs for onshore wind projects, according to IRENA 2021 data vary by location. Generally, in 2020 however, the O&M costs ranged between 10 and 30% of the LCoE for the majority of projects. Applying those percentages to the total investment cost, at the country level, between 2016 and 2019, O&M costs for onshore wind projects ranged from 33 €/kW per year in Denmark to 56 €/kW per year in Germany^20^. Nonetheless, investigations from several reputable sources have shown that O&M costs for onshore wind projects, in terms of cost per kWh, were between 0.01 and 0.02 €/kWh over the lifetime of the wind farm^23^. Since in Albania all onshore wind farm perspectives implementations are in the phase of collecting wind speed measurements on the ground, we do not have any data indicative of the O&M value. For this reason, in our research work, for both proposed parks O&M was assumed to be 0.018 €/kWh over the lifetime of the wind turbine or 55 €/kW of installed power.

### Methodology for wind energy cost analysis

The main methods that represent the indicators for determining the efficiency and technical–economic character of energy systems in general and wind energy systems in particular were analyzed in this section.

*Levelized cost of electricity* (*LCoE*) is an energy source measuring scale that has been used to compare the different methods of energy production through comparable criteria. This would be cost information for the investor to produce one kWh of electricity produced by a certain technology. LCoE can be calculated by using Eq. (2).

2 $${ \rm ``} LCoE {\rm "}= \frac{NPV \,\,of\,\, total \,\,costs\left(\text{\EUR}\right)}{Total \,\,energy \,\,production \left(kWh\right)}=\frac{{\sum }_{t=1}^{n}\frac{{I}_{t}+{O\&M}_{t}+{F}_{t}}{{\left(1+r\right)}^{t}}}{{\sum }_{t=1}^{n}{E}_{t}}(\text{\EUR}/{\text{kWh}})\mathrm{ or }(\text{\EUR}/{\text{MWh}}).$$

Where *n* is the lifetime of the energy farm in which electricity is generated in years, *E*_*t*_ is the annual energy production (AEP) in the year *t* (kWh) or (MWh), *I*_*t*_ is CAPEX in year *t* (€), O&M_t_ is operation and maintenance cost (OPEX) in year *t* (€), *F*_*t*_ is fuel expenditures in the year *t* (€), and *r* is the discount rate^26–28^.

The levelized cost of electricity of onshore wind farms in Europe, according to the Bloomberg New Energy Finance data, in 2018 ranges from 50 € to 65 €/MWh^29^.

The *Discount rate* (*r*) is used to bring future costs back to their present value. We predict the project yearly income to remain the same during a 20-year project lifetime. However, due to inflation, this income would not have the same value in the future as it does now. The discount rate is used to calculate the decline in value of money in the future. This financial method is known as the discount cash flow.

Discount rate values vary depending on the technology of the investment projects. Many studies analyzing wind energy project cost elements, based on the evidence gathered from the literature review, show that the range of discount rates for onshore wind energy projects varies between 7 and 10%, directly affecting the *LCoE* value.

Several factors increase the perception of risk for wind energy technology investments including wholesale electricity prices, changes in government policy and less mature technology. The discount rate range for onshore projects is predicted to be between 5 and 8% in the year 2040. This takes into account the importance that renewable energy technologies are gaining currently not only due to the energy crisis but also to the 1.5 ^0^C climate target under the Paris Agreement. On the other hand, this will be paired with high expectations of improved wind technology maturity in the future. In this study the discount rate was assumed to be 5, 7 and 11%.

The *net present value* (*NPV*), gives us the ability to estimate whether or not to move forward with the project. The total project yearly cash flow equals the given year’s income (B_i_) minus that year’s costs *C*_*i*_. But this yearly project cash flow is expressed in today’s value of money. Then we subtract the initial capital investment cost *C*_*0*_ from the total cash generated during the planned lifetime of the project *N* to obtain the next present value of the project. This is shown in Eq. (3). If the net present value is positive then the project is feasible and suitable for further consideration^30^.

3 $$NPV=\sum_{i=1}^{N}\frac{{B}_{i}-{C}_{i}}{{\left(1+r\right)}^{i}}-{C}_{0}, [\text{\EUR}].$$

*Internal rate of return* (*IRR*) is considered to be the discount rate for which the Net Present Value (NPV) of the project is zero. This is expressed in Eq. (4).

4 $$0= \sum_{n=0}^{N}\frac{{C}_{n}}{{\left(1+IRR\right)}^{n}}$$

where *N* is the lifetime of the project in years, and *C*_*n*_ is the cash flow for year *n*. An investment is considered acceptable if it’s *IRR* is greater than the investor’s minimum acceptable rate of return.

The *Benefit–Cost ratio* (*B–C*) is an expression of the relative profitability of the project. It is calculated as a ratio of the present value of annual revenue minus the annual costs to the project equity which is expressed by Eq. (5).

5 $$B-C=\frac{NPV+\left(1-{f}_{d}\right)C}{\left(1-{f}_{d}\right)C}$$

where *f*_*d*_ is the debt ratio. The greater the value of this ratio, the bigger the return on investment in either revenue or savings terms. Obviously in order for the investment to be considered economically efficient this ration needs to be greater than one.

*Simple payback time* refers to the number of years necessary for the project revenue by excluding debt payments and is equal to the total investment cost. While Equity payback indicates the years after which the project will generate income beyond the initial investment value.

### Research work proposal cost

The RETScreen Expert model was used to perform the financial analysis and determine the aforementioned financial parameters *LCoE, NPV, IRR, B-C*, and *SPB* for the research work proposed in “Vajkal”, Bulqizë and “Selitë e Malit”, Tirana county. RETScreen Expert is a clean energy management software used for energy efficiency, renewable energy, energy performance analyses and environmental impact. Via sensitive analyses, this program quickly allows professionals to determine whether a proposed renewable energy research work contains financial parameters suitable to move it forward. Therefore, the RETScreen software is a great and user-friendly tool for analyzing the feasibility of renewable energy projects since it can estimate energy production, entire life cycle cost, and the greenhouse gas emission reductions resulting from implementing the project.

In order to perform the financial analysis for the proposed projects, the model requests average monthly wind speed data as well as other climate data such as average air temperature, humidity, atmospheric pressure, etc. RETScreen referred to the climate data obtained from the National Aeronautics and Space Administration (NASA) regarding the geographic location of the antennas. Meanwhile, average monthly wind speed data was manually inputted in accordance to the NEWA hypothetical antennas in the 2005–2018 timeframe. Table 5 depicts the main tecno-economic components considered by RETScreen to conduct the financial analysis for the selected areas “Vajkal” and “Selite e Malit”.

**Table 5:** The main technical and economic parameters for the proposed research work in “Vajkal” and “Selitë e Malit”.

Components	Value	Unit
Installed capacity	9	MW
Number of V100-1.8 wind turbines	5	Pcs
Wind speed—annual, Vajkal/Selitë e Malit	6/5.3	m/s
Availability	97	%
Electricity export rate	76	€/MWh
Investment cost	1350	€/kW
Discount rate	7	%
Debt ratio	70	%
Debt interest rate	3	%
Debt term	15	Year
Inflation rate	3	%
Project life	20	Year
(O&M) cost	0.018	€/kWh
Land lease	Not applicable	–

Based on the tecno-economic data in Table 5, the RETScreen Expert model calculated the financial indicators presented in Table 6.

**Table 6:** Financial indicators for both of proposed wind farms based on parameters given in Table 5.

Financial indicators	Vajkal wind farm		Selitë e Malit wind farm	
	From WAsP	From RETScreen	From WAsP	From RETScreen
Annual energy production (MWh)	31,571	26,336	28.909	23,716
Capacity factor (%)	40	33.4	36.6	30.1
LCoE, (€/kWh)		0.061		0.067
NPV, (€)		8,221,399		5,718,811
Benefit–cost (B–C) ratio		3.3		2.6
Pre-tax IRR—equity, (%)		25.4		19.9
Simple payback, (year)		8.1		9.3
Equity payback, (year)		4.2		5.5

The difference between the annual energy production of the proposed parks calculated by WAsP and RETScreen are explained by the fact that RETScreen ran with average annual wind speed values from the NEWA hypothetical antennas which unlike the WAsP turbines were not positioned in the highest potential. The mean annual wind speed for the locations of the Vajkal and Selitë e Malit turbines from WAsP were found to be respectively 6.72 m/s and 6.81 m/s. Hence this difference is to be expected given that energy production is proportional to wind speed cubed.

### Sensitivity and risk analysis

**Figure 11:**
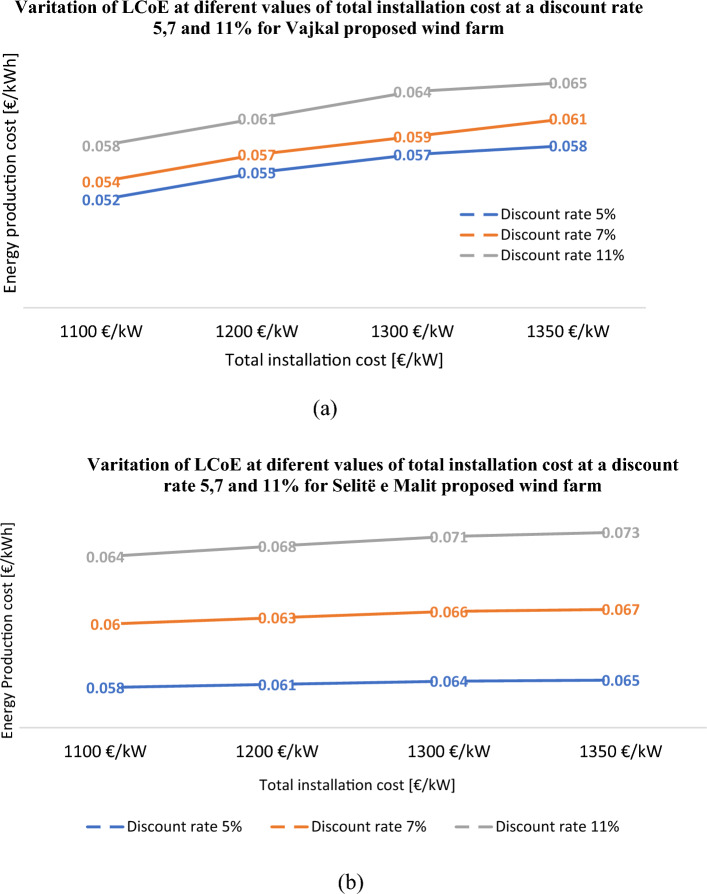
*LCoE* variation as a function of total installation cost (€) and discount rate (%) for electricity export rate 76 €/MWh. (**a**) “Vajkal” and (**b**) “Selitë e Malit” wind farm.

An important parameter that helps determine whether to produce wind energy is the energy production cost *LCoE* of the proposed wind farms. Figure 11 depicts *LCoE* as a function of the different values of total installation cost for discount rates 5, 7, and 11% and electricity export rate 76 €/MWh for the “Vajkal” and “Selitë e Malit” wind farms.

**Figure 12:**
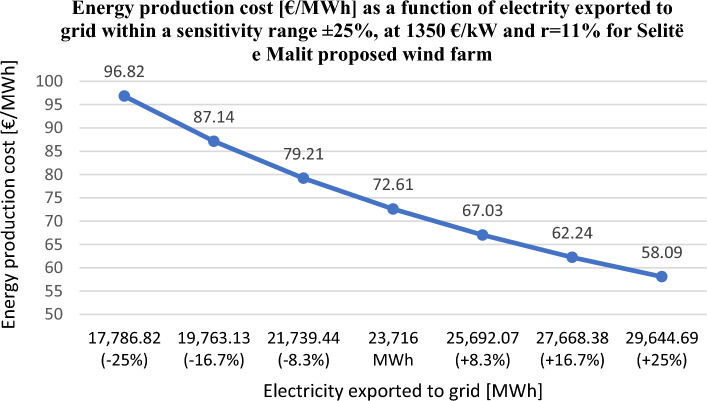
*LCoE* variation as a function of annual electricity production within a sensitivity range ± 25%, at total installation cost 1350 €/kW and at 11% discount rate for “Selitë e Malit” wind farm.

From the Fig. 11 it has been clearly shown that the *LCoE* for the park proposed in “Vajkal” is similar to European wind farms for every discount rate value and for a total installation cost of 1350 €/kW. For the wind farm proposed in “Selitë e Malit”, the variation of *LCoE* as a function of annual energy production within a sensitivity range of ± 25%, at 1350 €/kW installation cost and at 11% discount rate, is given in Fig. 12.

**Figure 13:**
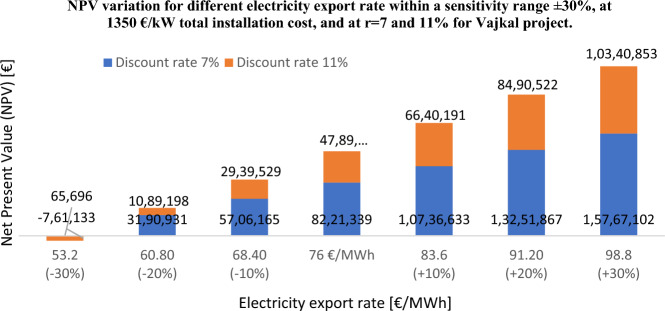
*NPV* variation as a function of electricity export rate within a sensitive range ± 30%, at 1350 €/kW total installed cost and at *r* = 7, 11% for Vajkal proposed wind farm.

Furthermore, in this sensitivity analysis it is observed that by increasing the annual electricity production by at least 9%, even when the proposed research work is perceived to be high risk, the LCoE calculated by the RETScreen model will be within the European wind farm values. Hence, we can consider the annual energy production calculated by WAsP, both proposed parks are feasible in terms of *LCoE*. Investors would be interested to understand what happens to the other important financial indicator such as *NPV* if the electricity export rate changes within a sensitivity range of ± 30%, assuming a total installation cost of 1350 €/kW, at 7 and 11% discount rates, see Fig. 13.

Through a total installation cost of 1350 €/kW, an inflation rate of 3%, a debt rate of 70%, a debt term of 15 years, and an electricity export rate within a range of ± 30%, it is observed that the *NPV* calculated at 7 and 11% discount rate reduces by a factor of 2.5 and 4 respectively if an electricity export rate of 60.80 €/MWh is assumed. For electricity export rates smaller than this value, the NPV declines significantly, sliding even into negative values. In this case the proposed “Vajkal” park is considered infeasible. For the park proposed in “Selite e Malit”, at the same parameters mentioned above, an electricity export rate would be no smaller than 70 €/MWh. This price can go down to 63.3 €/MWh if the total installation cost declines to 1125 €/kW.

**Figure 14:**
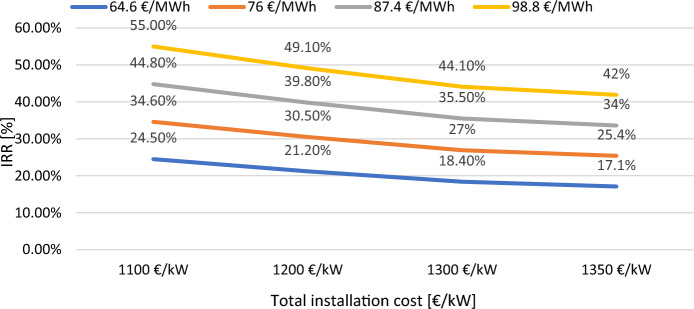
*IRR* variation as a function of total installation cost and electricity export price for the proposed park in Vajkal.

In the Fig. 14 it has been shown *IRR* as a function of the total installation cost (1100, 1200, 1300, and 1350 €/kW) and electricity export rate within a sensitivity range of ± 30%, calculated for a debt rate of 70% and inflation rate of 3%. The discount rate does not have an effect on *IRR*. As it can be seen, the lower the total installation cost is, the higher the IRR values. For an electricity export rate of 64.6 and 76 €/MWh, which actually is the electricity price benchmark, the *IRR* values for the project proposed in “Vajkal” are optimal throughout the entire range of electricity export rates in the graph.

Another financial indicator is the Benefit–Cost ratio (B–C). *B–C* is an expression of the relative profitability of the project. *B–C* variation as a function of total installation cost and discount rate for 76 €/MWh for “Vajkal” and “Selitë e Malit” parks, is given in Table 7.

**Table 7:** *B–C* variation as a function of total installation cost and discount rate for 76 €/MWh for “Vajkal” and “Selitë e Malit” parks.

Electricity export rate (€/MWh)	76												
Vajkal wind farm, Bulqiz	Discount rate (%)	5	5	5	5	7	7	7	7	11	11	11	11
	Total installation cost (€/kW)	1100	1200	1300	1350	1100	1200	1300	1350	1100	1200	1300	1350
	B–C ratio	5.3	4.7	4.2	4	4.4	3.9	3.4	3.3	3.2	2.8	2.5	2.3
	Equity payback (years)	3	3.5	4	4.2	3	3.5	4	4.2	3	3.5	4	4.2
Selitë e Malit wind farm, Tirana	B–C ratio	4.3	3.8	3.3	3.1	3.6	3.1	2.7	2.6	2.5	2.2	**1.9**	**1.8**
	Equity payback (years)	3.8	4.4	**5.1**	**5.5**	3.8	4.4	**5.1**	**5.5**	3.8	4.4	**5.1**	**5.5**

Sensitivity and risk analysis for “Selitë e Malit” park with the values highlighted in bold has shown that a total installation of 1300 and 1350 €/kW at discount rate 11% corresponds to the lower values B–C ratio values. However, the values of 1300 and 1350 €/kW at equity payback will not been acceptable and therefore the proposed research work in this case will not be feasible.

### Environmental impact

The technology of producing electricity from wind energy does not emit greenhouse gases in the environment whereas the conventional technologies in energy production contributed 78% of total EU emissions^31–33^. The electricity produced in Albania comes 100% from renewable energies. Since Albania is a net importer of energy, it is forced, depending on the hydrological conditions, to import energy from the region and mainly from Kosovo. Coal is the most important energy source of Kosovo, from which about 97% of electricity is produced. Therefore, we can say that every MWh of energy produced by wind energy in Albania would contribute to the decarbonization of the energy sector in the region^34–38^. To calculate the contribution of the electricity produced by the two proposed parks to the reduction of CO_2_ was used RETScreen Greenhouse Gas (GHG) Emission Reduction Analysis model. Scenarios were assumed as if this electricity was produced by coal-fired power plants in Kosovo, or by those with natural gas under the conditions of the parameters of the power plants in Germany. RETScreen model calculates the amount reduced in tCO_2_ and equates it to barrels of crude oil not consumed.

**Figure 15:**
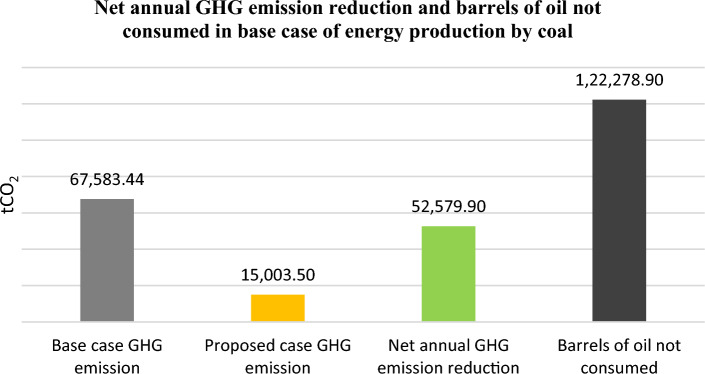
GHG analysis of the proposed “Vajkal” and “Selitë e Malit” wind farms and benefits in tCO_2_ reduction with a coal power plant as base case.

Figure 15 depicts the annual amount in tCO_2_ that is reduced by producing electricity from the proposed parks in “Vajkal” and “Selitë e Mali” and is compared to the base case if this energy would be produced from coal.

**Figure 16:**
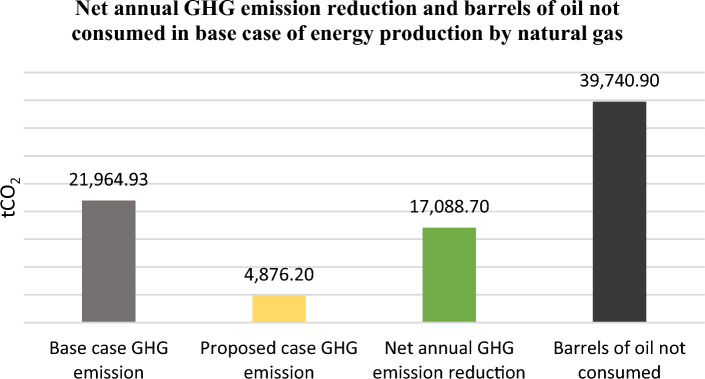
GHG analysis of the proposed “Vajkal” and “Selitë e Malit” wind farms and benefits in tCO2 reduction with a natural gas plant as base case

In Fig. 16, the base case that will replace the wind energy production is a natural gas plant. In this case, it will be reduced every year for 20 years of wind farms lifetime, the emission of 17,088.7 tCO_2_ which is equivalent to 39,740.9 barrels of oil not consumed.

## Conclusions

This research work demonstrated the importance of implementing renewable energy in the metallurgical sector in Albania. It presented the possibility of implementing wind energy in the metallurgical companies that operate in Albanian for production of steel, aluminum and chromium. The Wind Balkan Atlas (WBA) and New European Wind Atlas (NEWA) were used to select the appropriate areas, while the Wind Atlas Analysis and Application Program (WAsP) was used to develop the wind potential distribution maps, and select the most suitable type of wind turbine based on capacity factors. The results show two suitable areas selected close to metallurgical sectors in the regions of “Vajkal” in Bulqizë and “Selitë e Malit” in Tirana. The selected areas can be classified as wind power class IV, V and VI. Vestas wind turbines type V100-1.8 has been selected and five wind turbines have been implemented for each area to maximizes annual electric power production and minimizes wake losses.

It has been installed the power of 9 MW for each wind farm, with a capacity factor of 40% and 36.6% respectively, and with a total annual energy production of about 60 GWh/year, these wind farms will cover about 26% of the total annual consumption of companies.

RETScreen Expert was used for the detailed economic analysis and environmental impact of proposed wind farms. The economic sensitivity analysis of the proposed wind farm in “Vajkal” showed that even for the highest installation cost value of 1350 €/kW, for discount rates 5, 7, and 11%, the *LCoE* values are within the statistically established range for wind farms in Europe. On the other hand, the wind farm in “Selite e Malit” would require an annual electricity production increase of at least 9% so that even when the project is considered at the highest risk level, the *LCoE* is still within established values. However, keeping in mind the annual energy production calculated from WAsP for optimizing the position of wind turbines, we can conclude that both wind farms proposed are feasible in terms of *LCoE*.

From the implementation of the two proposed parks every year for 20 years of the wind farm lifetime of the parks, the emission of 52,579.9 tCO_2_ and 17,088.7 tCO_2_ will be reduced, respectively if this amount of energy will be produced by a coal or natural gas power plant.

These wind farms will play an important role in the context of industrial energy communities in Albania. All the wind farms will serve as primary and supplementary source of energy by providing clean and renewable electricity in the whole industrial communities**.** In any energy crises these wind farms implementation can help these companies to avoid the import of electricity and increasement of the production prices of iron, steel, chromium and aluminum. Most of these industrial companies will not be dependent on non-renewable sources of energy and giving contribution to a more sustainable energy infrastructure.

The study of the fluctuation of energy produced by wind farms and the profile over time of energy consumption by companies is an aspect of wind energy modeling for these industries. However, in the conditions of Albania, since wind energy is well-balanced with hydroelectric energy, diversifying energy sources will increase supply security. The future research work will be focused on continuing energy efficiency analysis through optimization process in the metallurgy sector by starting from materials flows, waste heat recovery, energy-efficient lighting and equipment.

## Data Availability

The data that support the findings of this study are available from the corresponding author upon request.

## Abbreviations

AEP: Annual energy produced
B-C: Benefit–cost ratio
BWA: Balkan Wind Atlas
CAPEX: Capital expenditures
EWEA: European Wind Energy Association
GIS: Geographic Information System
IEA: International Energy Agency
IRENA: International Renewable Energy Agency
IRR: Internal rate of return
LCoE: Levelized cost of electricity
MERRA: Modern-Era Retrospective Analysis for Research and Applications
NASA: National Aeronautics and Space Administration
NEWA: New European Wind Atlas
NPV: Net present value
OPEX: Operation and maintenance cost
OWC: Observed wind climate
RETScreen: Clean energy management software
UTM: Universal Transverse Mercator
WAsP: Wind Atlas Analysis and Application Program
