# Publisher Correction: Modelling the wind potential energy for metallurgical sector in Albania

**DOI:** 10.1038/s41598-024-55388-9

**Published:** 2024-02-27

**Authors:** Klodian Dhoska, Elena Bebi, Irida Markja, Parid Milo, Ermil Sita, Serxhi Qosja

**Affiliations:** 1 Department of Production and Management, Faculty of Mechanical Engineering, Polytechnic University of Tirana, Square Mother Theresa No. 1, 1019 Tirana, Albania; 2 Albanian Mechanical Engineering Association (AMEA), Mother Theresa Square No. 1, 38954 Tirana, Albania; 3 Association of Talent Under Liberty in Technology (TULTECH), Sopruse pst 3, 10615 Tallinn, Estonia; 4 Department of Energy, Faculty of Mechanical Engineering, Polytechnic University of Tirana, Square “Mother Theresa” No. 1, 1019 Tirana, Albania; 5 Department of Mechanics, Faculty of Mechanical Engineering, Polytechnic University of Tirana, Square “Mother Theresa” No. 1, 1019 Tirana, Albania; 6 Everest Ltd, Metallurgical Industry, Bulevardi Blu 544, Kamëz, 1030 Tirana, Albania

Correction to: *Scientific Reports* 10.1038/s41598-024-51841-x, published online 14 January 2024

The original version of this Article contained errors.

Affiliation 1 was incorrectly given as ‘Department of Production and Management, Faculty of Mechanical Technology, Polytechnic University of Tirana, Square Mother Theresa No. 1, 1019, Tirana, Albania’. The correct affiliation is listed below.

Department of Production and Management, Faculty of Mechanical Engineering, Polytechnic University of Tirana, Square Mother Theresa No. 1, 1019, Tirana, Albania

Affiliation 4 was incorrectly given as ‘Department of Energy, Faculty of Mechanical Technology, Polytechnic University of Tirana, Square “Mother Theresa” No. 1, 1019, Tirana, Albania’. The correct affiliation is listed below.

Department of Energy, Faculty of Mechanical Engineering, Polytechnic University of Tirana, Square “Mother Theresa” No. 1, 1019, Tirana, Albania

Affiliation 5 was incorrectly given as ‘Department of Mechanics, Faculty of Mechanical Technology, Polytechnic University of Tirana, Square “Mother Theresa” No. 1, 1019, Tirana, Albania’. The correct affiliation is listed below.

Department of Mechanics, Faculty of Mechanical Engineering, Polytechnic University of Tirana, Square “Mother Theresa” No. 1, 1019, Tirana, Albania

In addition, the legends of Figure 1 to Figure 15 were inadvertently switched. As a result,

The legend of Figure 1:

“The flowchart of the feasibility study for setting up wind farms in the selected areas.”

now reads:

“Energy consumption profile for the steel, ferrochrome and aluminum companies, located in Albania.”

The legend of Figure 2:

“The selected areas in the map of wind speed distributions at 50 m a.g.l. in the Albania’s territory from both atlases: (**a**) Wind Balkan Atlas and (**b**) New European Wind Atlas.”

now reads:

“The flowchart of the feasibility study for setting up wind farms in the selected areas.”

The legend of Figure 3:

“The selected areas in the map of wind speed distributions at 50 m a.g.l. from Wind Balkan Atlas: (**a**) “Vajkal”, Bulqizë and (**b**) Selitë e Malit, Tirana county.”

now reads:

“The selected areas in the map of wind speed distributions at 50m a.g.l. in the Albania’s territory from both atlases: (**a**) Wind Balkan Atlas and (**b**) New European Wind Atlas.”

The legend of Figure 4:

“The mutual positions of the anemometric towers according to the two atlases: (**a**) in the area of “Vajkal”, Bulqiza and (**b**) “Selitë e Malit”, Tirana county.”

now reads:

“The selected areas in the map of wind speed distributions at 50m a.g.l. from Wind Balkan Atlas: (**a**) “Vajkal”, Bulqizë and (**b**) Selitë e Malit, Tirana county.”

The legend of Figure 5:

“The white line shows the 110 kV voltage line and the Peshkopi-Maqellare national road that passes near the “Vajkali” area.”

now reads:

“The mutual positions of the anemometric towers according to the two atlases: (**a**) in the area of “Vajkal”, Bulqiza and (**b**) “Selitë e Malit”, Tirana county.”

The legend of Figure 6:

“Wind rose charts and Weibull probability distributions for two selected areas at 50 m above ground level through WAsP. “Vajkal”, Bulqizë according to (**a**) BWA and (**b**) NEWA. “Selitë e Malit”, Tirana county according to (**c**) BWA and (**d**) NEWA.”

now reads:

“The white line shows the 110 kV voltage line and the Peshkopi-Maqellare national road that passes near the “Vajkali” area.”

The legend of Figure 7:

“Wind potential distribution maps in selected areas at 50 m above ground level through WAsP. “Vajkal”, Bulqizë according to (**a**) wind speed and (**b**) power density. “Selitë e Malit”, Tirana county according to (**c**) wind speed and (**d**) power density.”

now reads:

“Wind rose charts and Weibull probability distributions for two selected areas at 50 m above ground level through WAsP. “Vajkal”, Bulqizë according to (**a**) BWA and (**b**) NEWA. “Selitë e Malit”, Tirana county according to (**c**) BWA and (**d**) NEWA.”

The legend of Figure 8:

“Capacity factors in the percentage for four different types of Vestas wind turbines.”

now reads:

“Wind potential distribution maps in selected areas at 50 m above ground level through WAsP. “Vajkal”, Bulqizë according to (**a**) wind speed and (**b**) power density. “Selitë e Malit”, Tirana county according to (**c**) wind speed and (**d**) power density. *Source: Author’s design.*”

The legend of Figure 9:

“General view of proposed wind farms. (**a**) “Vajkal”, proposed wind farm and (**b**) “Selitë e Malit” proposed wind farm.”

now reads:

“Capacity factors in the percentage for four different types of Vestas wind turbines tested in Vajkal, Bulqizë site.”

The legend of Figure 10:

“*LCoE* variation as a function of total installation cost (€) and discount rate (%) for electricity export rate 76 €/MWh. (**a**) “Vajkal” and (**b**) “Selitë e Malit” wind farm.”

now reads:

“General view of proposed wind farms. (**a**) “Vajkal”, proposed wind farm and (**b**) “Selitë e Malit” proposed wind farm.”

The legend of Figure 11:

“*LCoE* variation as a function of annual electricity production within a sensitivity range ± 25%, at total installation cost 1350 €/kW and at 11% discount rate for “Selitë e Malit” wind farm.”

now reads:

“*LCoE* variation as a function of total installation cost (€) and discount rate (%) for electricity export rate 76 €/MWh. (**a**) “Vajkal” and (**b**) “Selitë e Malit” wind farm.”

The legend of Figure 12:

“*NPV* variation as a function of electricity export rate within a sensitive range ± 30%, at 1350 €/kW total installed cost and at *r* = 7, 11% for Vajkal proposed wind farm.”

now reads:

“*LCoE* variation as a function of annual electricity production within a sensitivity range ±25%, at total installation cost 1350 €/kW and at 11% discount rate for “Selitë e Malit” wind farm.”

The legend of Figure 13:

“*IRR* variation as a function of total installation cost and electricity export price for the proposed park in Vajkal.”

now reads:

“*NPV* variation as a function of electricity export rate within a sensitive range ±30%, at 1350 €/kW total installed cost and at *r* = 7, 11% for Vajkal proposed wind farm.”

The legend of Figure 14:

“GHG analysis of the proposed “Vajkal” and “Selitë e Malit” wind farms and benefits in tCO_2_ reduction with a coal power plant as base case.”

now reads:

“*IRR* variation as a function of total installation cost and electricity export price for the proposed park in Vajkal.”

The legend of Figure 15:

“GHG analysis of the proposed “Vajkal” and “Selitë e Malit” wind farms and benefits in tCO_2_ reduction with a natural gas plant as base case.”

now reads:

“GHG analysis of the proposed “Vajkal” and “Selitë e Malit” wind farms and benefits in tCO_2_ reduction with a coal power plant as base case.”

Finally, the legends of Table 2 and Table 3 were incorrect.

The legend of Table 2:

“Mean wind speed (m/s) and power density (W/m^2^) at 50 m a.g.l. in selected areas.”

now reads:

“Mean wind speed (m/s) and power density (W/m^2^) at 50 m a.g.l. in selected areas according to data of period time 2005-2018 from NEWA.”

The legend of Table 3:

“The main characteristics of the selected wind turbines.”

now reads:

“The main characteristics of the selected wind turbines. *Source: WAsP wind turbine files.*”

The original Article has been corrected.

